# Copolymers of NVAm and NVP for Efficient Gene Delivery

**DOI:** 10.1021/acspolymersau.5c00167

**Published:** 2026-01-16

**Authors:** Tom Fielitz, Christopher Raab, Vitalii Tkachenko, Kristine M. Oleszkiewicz, Hendrik Fuchs, Matthias Hartlieb

**Affiliations:** a Institute of Chemistry, University of Potsdam, Karl-Liebknecht-Straße 24-25, Potsdam DE-14476, Germany; b Institute of Diagnostic Laboratory Medicine, Clinical Chemistry and Pathobiochemistry, 14903 Charité − Universitätsmedizin Berlin, corporate member of Freie Universität Berlin and Humboldt-Universität zu Berlin, Augustenburger Platz 1, Berlin DE-13353, Germany; c Fraunhofer Institute for Applied Polymer Research IAP, Geiselbergstraße 69, Potsdam 14476, Germany; d Institute of Pharmacy, Freie Universität Berlin, Königin-Luise-Straße 2 and 4, Berlin DE-14195, Germany; e Institute of Chemistry and Biochemistry, Freie Universität Berlin, Arnimallee 20, Berlin DE-14195, Germany

**Keywords:** photoiniferter RAFT polymerization (PI-RAFT), *N*-vinyl formamide, polyamine, charge density control, transfection optimization, gene delivery vectors

## Abstract

Gene delivery lies at the heart of many approaches for treating a host of different diseases. Promising candidates for the delivery of genetic material are polycationic vectors; however, managing toxicity arising from adverse interactions with the lipid bilayer remains a challenge. In this work, photoiniferter reversible addition–fragmentation chain-transfer (PI-RAFT) polymerization was used to synthesize statistical copolymers of *N-*vinyl formamide (NVF) and *N*-vinyl pyrrolidone (NVP). Subsequent selective hydrolysis of NVF was used to introduce polyvinyl amine (PVAm) repeats. The resulting library of polymers with varying charge densities and molar masses was probed for biocompatibility with erythrocytes and MDA-MB-468 cells, revealing substantially reduced cytotoxicity compared with linear polyethylene imine (lPEI) and Lipofectamine 2000. Using an ethidium bromide (EtBr) replacement assay, PVAm copolymers were shown to replace EtBr at low N/P-ratios. The transfection conditions were optimized in terms of the N/P-ratio and polyplex concentration by a *Renilla* luciferase reporter assay. This revealed 30-fold less cytotoxicity, a much wider viable concentration range, and a 2-fold greater transfection efficiency for the PVAm copolymer compared to lPEI. This study provides insights into the PI-RAFT copolymerization of the less activated monomers NVF and NVP and highlights the potential of polyvinyl amine copolymers resulting from selective hydrolysis for the transfection of genetic material compared with lPEI.

## Graphical abstract

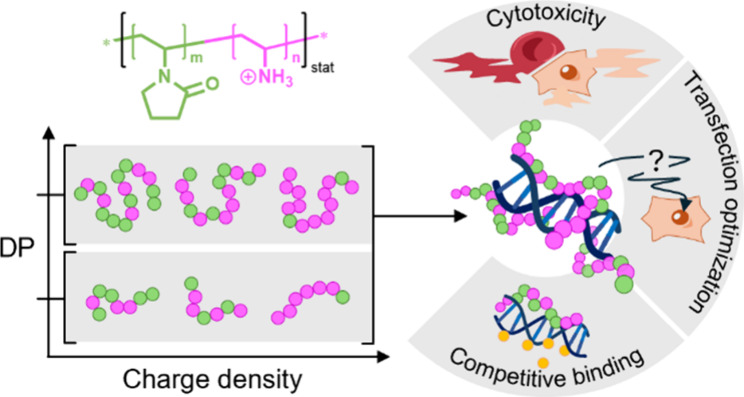

## Introduction

Pharmaceuticals based on genetic material hold great promise for the treatment of a variety of inherited and acquired diseases (e.g., hemophilia,^1^ hepatitis or cancer^2,3^ ). In the domain of cancer treatment, suicide gene therapy constitutes a pioneering approach that involves the introduction of a gene encoding a protein toxin or a prodrug-converting enzyme, thereby rendering the targeted cell more susceptible to chemotherapy.^4^ However, the key challenge for drugs that use nucleic acids (NAs) as active ingredients is enabling transport to and transfection into the desired cellular target. Naked NAs in circulation are prone to degradation by nucleases and exhibit overall low transfection efficacy due to repulsive interactions with equally negatively charged cell membranes.^3^ To overcome this issue and enable effective gene transfer, vectors based on viruses,^5^ liposomes, lipid nanoparticles,^6^ or polymers^7,8^ can be used. Each method is characterized by its own set of benefits and shortcomings. While viral transfection agents benefit from evolutionarily acquired high transfection efficacies and possible targeted selectivity by altering surface proteins, their manufacturing process is tedious, and strong immune responses are possible, impacting treatment decisions.^5,9,10^ Lipid-based carriers have successfully entered clinical use (e.g., in recent mRNA-based COVID-19 vaccines^11,12^ ) and have demonstrated great potential as alternatives to viral vectors for a less pronounced immune response.^13^ However, limitations in gene-loading capacity, toxicity, and demand for specific storage conditions still need to be addressed.^14^ Stability problems include Ostwald ripening, drug leakage and aggregation. In addition, the manufacturing process is sophisticated, and special tools and expertise are required for steps such as high-pressure homogenization and microemulsion.^15^

Polymeric vectors contain cationic subunits that are able to form polyinterelectrolyte complexes (polyplexes) with NAs via electrostatic interactions. On the one hand, this prevents degradation, thus increasing the circulation time; on the other hand, it facilitates cellular uptake due to the higher membrane affinities of the positively charged polymer and polyplex.^3^ However, the inherent cytotoxicity of polycations must be addressed to balance desired and adverse effects, i.e., by altering the charge density^16^ or polymer topology.^17–19^

A prominent example of a polymer-based gene delivery vector is poly­(ethylene imine) (PEI), which is often considered the “gold standard” for polymeric vectors^20,21^ and is used in many studies as a reference. The structural characteristics of PEI are amenable to significant modification, thus increasing its biocompatibility, targeting, and delivery efficiency across a range of therapeutic platforms.^22^ The synthesis of structurally defined linear PEI requires precise conditions and high purity materials to effectively prevent side reactions.^23^ Polyvinyl amines (PVAm) – structural isomers of PEI, sharing its high charge density while possesing a purely carbon-based backbone – present themselves as alternative materials that can be produced under less demanding conditions. For copolymers incorporating PVAm, transfection efficacies rivaling PEI as well as lower cytotoxicity have been reported.^24–26^ Together with their easier purification and potentially lower cost,^24^ this makes VAm-containing polymers promising alternatives for the next generation of polymeric vectors.

The generation of PVAm is not straightforward due to the elusive nature of its monomer; vinyl amine tautomerizes to ethanimine (CH_3_CHNH), which readily forms trimers at room temperature,^27^ rendering direct polymerization impossible. Instead, a variety of precursors can be polymerized and chemically altered at a later step to reach the desired amine groups. Possible strategies include the polymerization of *N*-vinyl phthalimides and their hydrazinolysis,^28,29^ Hoffmann rearrangement of polyacrylamides^30^ and hydrolysis of poly­(*N*-vinyl amides).^17,18,31^ The use of *N*-vinyl formamide (NVF) is particularly promising because of its low cost as a monomer (used e.g., in the papermaking industry^32^), and the facile cleavage of the formyl group under relatively mild conditions,^33,34^ rendering this route compatible with other hydrolysis-labile groups and therefore enabling more complex molecular architectures.

One factor contributing to the recent investigations of PVAm for applications is surely the development of advanced techniques for reversible deactivation radical polymerization, allowing the synthesis of polymers from less activated vinylic compounds with low polydispersity and high control over molar mass.^35^ Guégan and co-workers reported the successful production of low dispersity PVAm (Đ ≤ 1.2) via organometallic-mediated radical polymerization (OMRP) of *N*-vinyl acetamide (NVA) and subsequent hydrolysis.^36^ Alternatively, reversible addition–fragmentation chain-transfer (RAFT) polymerization has been used by Destarac and co-workers for the preparation of polymers of NVF as well as NVA and its derivatives.^37,38^ Conventional RAFT can be simplified as free radical polymerization with the addition of a chain transfer agent (CTA), allowing reversible radical transfer and therefore much more homogeneous propagation. However, it still relies on exogenous initiators to provide the radicals required for polymerization. This may be considered an inherent disadvantage, as every introduced radical will produce a dead chain by irreversible termination.^39^ An alternative approach to this can be found in photoiniferter (PI)-RAFT polymerization. While this method shares the chain transfer step with the conventional RAFT process, radicals are generated by homolytic photolysis of the CTA, and recombination simply reforms a dormant chain, therefore decreasing the overall structural diversity of synthesized macromolecules.^40,41^ Coincidentally, xanthates, which are considered effective CTAs for the controlled polymerization of less activated monomers (e.g., NVF, NVA, vinyl acetate),^42^ are also comparatively efficient photoiniferters.^40,43^ However, there is very little coverage of the implementation of this method for vinyl amides, including only one report from Destarac and co-workers on the successful PI-RAFT polymerization of NVF and chain extension with *N*-vinyl pyrrolidone (NVP).^44^

In this work, we aim to shed more light on the ability of the PI-RAFT method for the statistical copolymerization of NVF and NVP at various ratios. Conditions for the selective hydrolysis of NVF repeating units to yield a P­(VAm-*co*-NVP) copolymer are investigated. The resulting materials are tested for their ability for gene transfection and toxicity to judge their potential as polymeric vectors, with an in-depth comparison to commercial linear PEI.

## Results and Discussion

### Polymer Synthesis

The polymers were synthesized via PI-RAFT polymerization with *O*-ethyl xanthate Xan as the chain transfer agent (Scheme 1). Xanthates are considered CTAs well suited for the RAFT polymerization of less activated monomers,^42^ and their ability to work in the successive PI-RAFT polymerization of NVF and NVP to produce block copolymers has been demonstrated as discussed earlier.^44^ To determine the optimal conditions for PI-RAFT polymerization, we ran several tests, synthesizing NVF homopolymers while varying the monomer concentration and irradiation intensity (Figure 1A and B). In all cases, conversions of approximately 80% can be reached upon prolonged irradiation of the mixture, with initial near linear regimes in first-order kinetic reaction plots. Initially, the rate of monomer conversion seems to be independent of monomer concentration, but the differences become more pronounced as the reaction reaches a plateau. As expected, the mixture viscosity increases significantly in correlation with the initial monomer concentration. The apparent stagnation of the reaction rate over time can likely be attributed to the destructive decomposition of the xanthate end-group. This is increasingly likely for mixtures with lower initial concentrations of xanthate, as the persistent radicals formed during photodissociation are less readily stabilized by dormant, undissociated xanthate species.^45^ As there is no additional radical source in the mixture, this side reaction reduces the number of radicals produced. The implications of this pathway are also apparent when examining the monomer conversion rate under various irradiation intensities (Figure 1B). While reactions conducted under high intensity initially proceed faster, they also reach a plateau much earlier, causing the polymerization to halt at lower maximum conversions. To maximize the polymer yield and achievable degree of polymerization (DP) while keeping the viscosity low for ease of handling, subsequent polymerizations were carried out at approximately 25 mW cm^–2^ and an initial monomer concentration of 2 mol L^–1^.

**Scheme 1:**
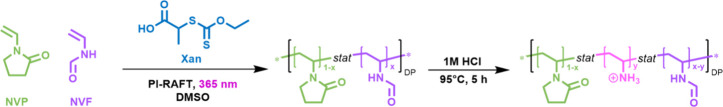
Simplified Reaction Scheme for the Synthesis of PVAm-Copolymers Discussed Herein Based on PI Polymerization of NVP and NVF and Subsequent Hydrolysis

**Figure 1:**
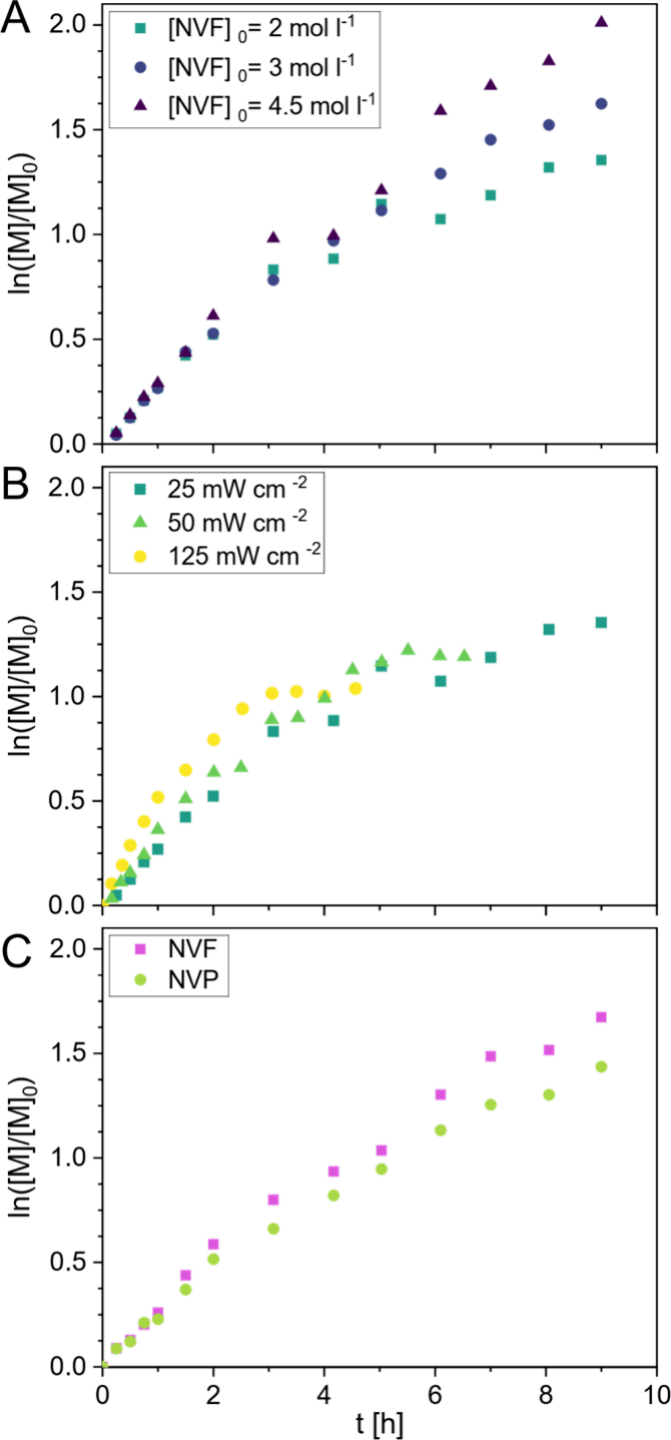
Polymerization kinetics under parameter variation. Conversion was determined via ^1^H NMR (details in the ). (A) Homopolymerization of NVF carried out in DMSO under constant irradiation (365 nm, 25 mW cm^–2^) and [NVF]_0_:[Xan]_0_ = 50:1 while varying the initial monomer concentration [NVF]_0_. (B) PI-RAFT homopolymerization of NVF carried out in DMSO at 365 nm with a constant initial monomer concentration of [NVF]_0_= 2 M and [NVF]_0_:[Xan]_0_ = 50:1 while varying the radiation intensity. (C) PI-RAFT copolymerization of NVF and NVP carried out in DMSO at [NVF]_0_ = [NVP]_0_ = 2.25 M, [NVF]_0_:[NVP]_0_:[Xan]_0_ = 25:25:1 under constant irradiation (365 nm, 25 mW cm^–2^).

To probe the PI-RAFT copolymerization of NVF and NVP, the individual conversion rates of both monomers in a mixture initially containing equal parts of NVF and NVP were tracked via isolated signals in the ^1^H NMR spectra (Figure S1). Over the course of the polymerization, NVF and NVP are consumed at almost equal rates, with a very slight preference for NVF (Figure 1C). This indicates the desired copolymerization behavior without apparent gradient formation.

On the basis of these results, two sets of polymers with different comonomer ratios and molar masses were produced (Table 1). They will further be denoted as short polymers or PXs (X being the mole fraction of NVAm repeating units as percentage) for the low molar mass set (entries 1–6) and long polymers or PXl (see above) for the high molar mass set (entries 7–12).

**Table 1:** Polymers Prepared via PI-RAFT Polymerization and Hydrolysis^a^

		Poly(vinyl amide)		Poly(vinyl amine)				
Polymer	[NVF]_0_:[NVP]_0_:[Xan]_0_	χ_NVF_ *(x)* in %	DP	Hydrolysis in %	X_NVAm_ *(y)* in %	*M*_n_in kg mol^–1^	Đ	#amines in mol kg^–1^
P82s^b^	44:6: 1.00	88	38	92	82	6.0	1.2	9.3
P75s^b^	40:10:1.00	80	37	95	75	7.4	1.4	8.2
P68s^b^	35:15:1.00	70	37	95	68	8.3	1.3	7.1
P54s^b^	30:20:1.00	60	35	92	54	9.3	1.3	5.7
P50s^c^	28:29:0.95	51	40	99	50	9.9	1.3	5.1
P41s^c^	24:36:0.83	42	38	98	41	9.3	1.3	4.0
P85l^d^	89:11:1.00	89	75	96	85	13.3	1.6	10.1
P76l^d^	78:22:1.00	79	79	97	76	11.5	1.8	8.6
P67l^d^	68:32:1.00	68	82	98	67	12.4	1.5	7.2
P59l^d^	59:41:1.00	60	86	>99	59	14.8	1.5	6.3
P49l^d^	49:51:1.00	49	89	>99	49	12.6	1.6	5.1
P39l^d^	39:61:1.00	39	90	>99	39	12.5	1.5	3.8

a PI-RAFT polymerizations carried out under irradiation at 365 nm (25 mW cm^–2^). Hydrolysis was carried out under acidic conditions (1 M HCl, 95 °C, 4 h).

b Polymerizations carried out at initial monomer concentration [NVF]_0_+[NVP]_0_ = 2 M, stopped after 9 h.

c Polymerizations carried out at [NVF]_0_+[NVP]_0_ = 2 M, stopped after 18 h.

d Polymerizations carried out at [NVF]_0_ = 1.8 M and under variation of [NVP]_0_ (0.2 M, 0.45 M, 0.77 M, 1.2 M, 1.8 M, 2.7 M), reactions stopped after 24 h.

The greater the amount of NVP present in the reaction mixture was, the lower the maximum conversion was even after prolonged reaction times. This behavior has been described just recently by Kwark and co-workers for the RAFT copolymerization of NVP and NVF.^46^ This might indicate a difference in tendencies for fragmentation in the RAFT equilibrium and should be investigated in future studies. To mitigate this effect and generate polymers with comparable chain lengths within a set, we probed two possible options. For the first set, the ratio of monomer to iniferter Xan [M]:[Xan] was increased, effectively increasing the targeted DP. For the second set, the overall monomer concentration was changed for each experiment to push back the onset of the reaction rate stagnation, as discussed earlier. With both approaches, the desired molar mass ranges could be achieved to a sufficient degree. However, the former approach – targeting higher DPs while keeping the overall monomer concentration constant – provided better control over monomer conversion. The resulting polymers are barely soluble in common solvents apart from water and DMSO. This is most likely due to the strong inter- and intramolecular H-bonding of the formamide repeating units, which have both donor and acceptor qualities. Unfortunately, this property prohibited us from subjecting them to standard SEC analysis for molar mass determination, as the polymers were not inert and hydrolyzed readily in the only aqueous elution system available, running at pH 2. Therefore, analysis of the molar mass for both sets was performed after the next reaction step and will be discussed below.

As discussed in the introduction, treating the polymers with acid or base allows cleavage of the formamide, revealing the desired primary amine (Scheme 1). This approach relies on the relative lability of the NVF-repeating unit’s formamide N-(C = O) bond compared with NVP’s N-(C=O) in the lactame ring. Hydrolysis of the latter would yield a zwitterionic species as a repeating unit bearing both secondary amine and carboxylic acid moieties. While investigating such structures may be interesting in the future, they are not the object of this study, making this process undesirable. Therefore, hydrolysis conditions mild enough to keep the lactame intact had to be found while still allowing near quantitative hydrolysis of the formamide repeating unit. This was achieved by dissolving the polymer in 1 M HCl and maintaining it at 95 °C for 5 h. To prove the stability of NVP, a homopolymer was produced via free radical polymerization and subjected to these conditions. The ^1^H NMR spectra taken of the isolated polymers before and after this trial showed no difference related to hydrolytic degradation (Figure S4).

The degree of hydrolysis of the polymers, which was monitored by ^1^H NMR (Figure S2), varies slightly between samples, with polymers containing a greater number of NVF repeating units showing a tendency toward incomplete cleavage. This is the case for both sets of polymers and is most likely related to charge repulsion. As the number of positively charged ammonium groups increases, the diffusion of H_3_O^+^ to the intact formamide units necessary for the protonation of neighboring amide bonds becomes increasingly challenging. Polymers containing more NVP allow charged units to be spaced out further owing to their statistical nature, therefore enabling higher degrees of hydrolysis. Similar observations and trends have been reported for the hydrolysis of NVF containing polymers before.^47,48^ The results of hydrolysis are highly reproducible, with deviations of less than 0.6% in the residual NVF content when multiple batches are prepared from the same virgin NVF-*co*-NVP copolymer (Figure S3).

After hydrolysis and purification, the polymers could be subjected to aqueous SEC and proved to be stable in the eluent. The molar mass and dispersity were determined via polyethylene glycol (PEG) calibration. Notably, the dispersity of the long polymers varies between 1.2 and 1.4, indicating reasonably good control of the PI polymerization over the less activated monomers. However, while elugrams remain monomodal, the dispersity of the high-DP set is significantly greater, indicating less favorable conditions and leaving room for improvement in further studies. The evident difference in molecular mass determined between samples with different amine contents might be the result of the PEG calibration failing to sufficiently model the hydrodynamic volume of the NVAm-*co*-NVP copolymers. Nonetheless, a distinct difference in the molar mass distributions in the chromatograms is evident between both sets of polymers.

The short polymers were subjected to potentiometric titration with 1 M NaOH (Figure 2B). The polymers do not exhibit distinct windows of buffering, as would be evident by pronounced plateaus (saddle points) in the protonation curve. This is to be expected for polycations, as the individual (de)­protonation equilibria of the amine groups are influenced by the groups in direct vicinity, and the structural diversity of the chemical environment in terms of chirality and polymer length imparts additional variation. Notably, this effect is less pronounced for polymers containing fewer amine groups, which is also evident in the first derivative plot, showing a more distinct local minimum. This is likely the case because the protonation equilibrium is more strongly influenced by other neighboring charged groups than by noncharged NVP repeating units.

**Figure 2:**
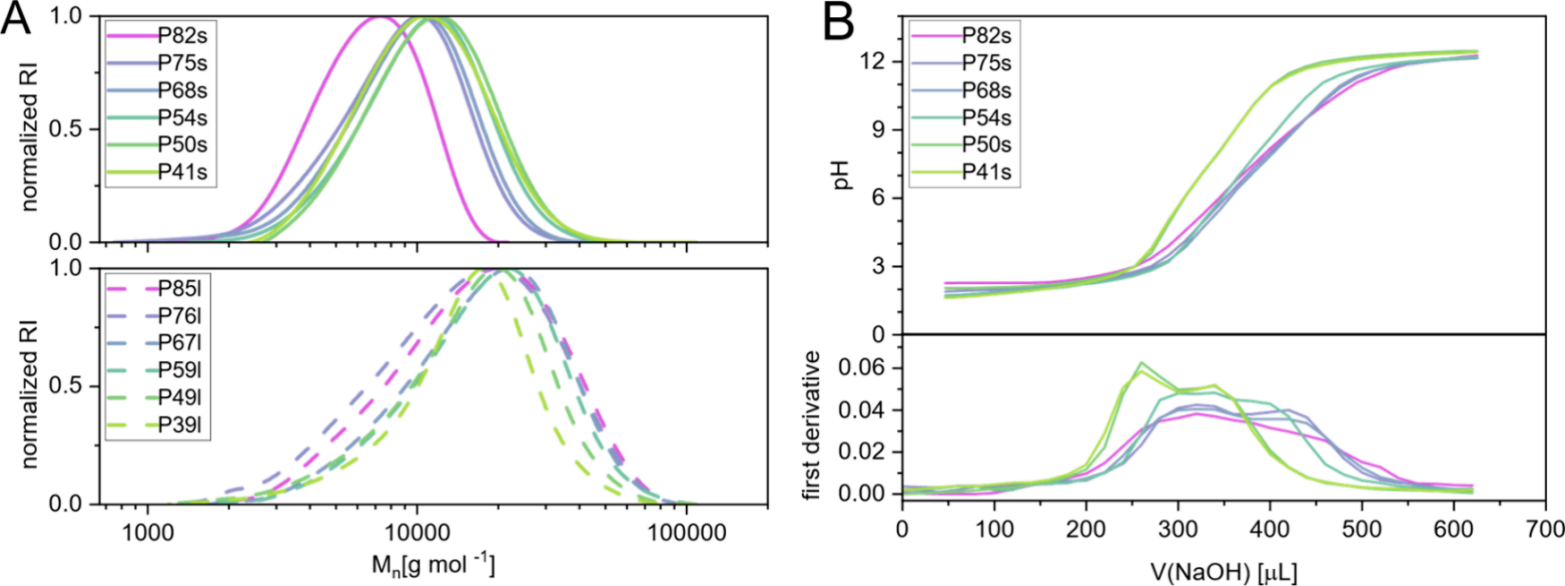
(A) SEC chromatograms of polyvinyl amines after hydrolysis measured in H_2_O (0.05 v% formic acid, 0.1 M HCl). A PEG standard was used for calibration. (B) Potentiometric titration of polymers with NaOH and first derivative of the curves.

### Biocompatibility

Our aim was to produce a polymer that is suitable for a broad range of transfection applications and could be used as a gene transfer vector. Since the cytotoxicity of PEI, which was used as a reference because of its widespread use,^20,21^ represents its most limiting property for the transfection of cells and in drug development in general,^21,49^ the effects of the polymers (Table 1) were initially probed for the viability of MDA-MB-468 cells, an aggressive breast cancer cell line.^50–52^ It is often used as a model in the early stages of research for breast cancer therapeutics and therefore represents a valuable model.^53,54^ For potential use as a gene transfer vector it is also imperative that the polymers do not interact negatively with blood components. Therefore, potential hematotoxicity was also investigated.

Both sets of polymers were tested for their biocompatibility by evaluating their lysis behavior against red blood cells and metabolic inhibition of the breast cancer cell line MDA-MB-468 via a 3-(4,5-dimethylthiazol-2-yl)-2,5-diphenyltetrazolium bromide (MTT) assay. For the MTT assay, linear PEI (lPEI, *M*_n_ = 25 kDa) and Lipofectamine (Lipo) 2000, a liposomal gene transfer agent, were used as controls. Both materials are commonly used vectors for the delivery of plasmids or small interfering RNAs into eukaryotic cells for protein expression or gene silencing. lPEI and Lipo 2000 are known for their high transfection efficiency but also exhibit high cytotoxicity.^21,55^ The low solubility of lPEI in the buffer medium prohibited comparative testing in hemolysis assays.

The concentrations at which cell viability was reduced to 50% in the MTT assay (IC_50_) and 10% of red blood cells were lysed (HC_10_) were derived by a logistical fit of the experimentally obtained data. Overall, higher values for these parameters indicate greater biocompatibility. The compounds exhibited similar trends in both assays. Generally, cell viability is lower for polymers with more amine groups. This is even more apparent for the long polymers, which have up to a 43-fold difference in IC_50_ compared with only up to a 5-fold difference for the short polymers (Figure 3). A potential underlying factor is the increased number of charged units per molecule which leads to greater enrichment of the anionic lipids of the cell membrane around the polymer. The resulting asymmetric distribution of phospholipids leads to increased permeability of the cell membrane.^56^ These trends illustrate the correlation of charge with toxicity and are in coherence with reported data on other polycations.^57^ However, all the compounds synthesized in this study have much higher IC_50_ values than Lipo 2000, ranging from a 23-fold increase to a 1000-fold increase, and lPEI, ranging from an 11-fold to a 500-fold increase in the IC_50_.

**Figure 3:**
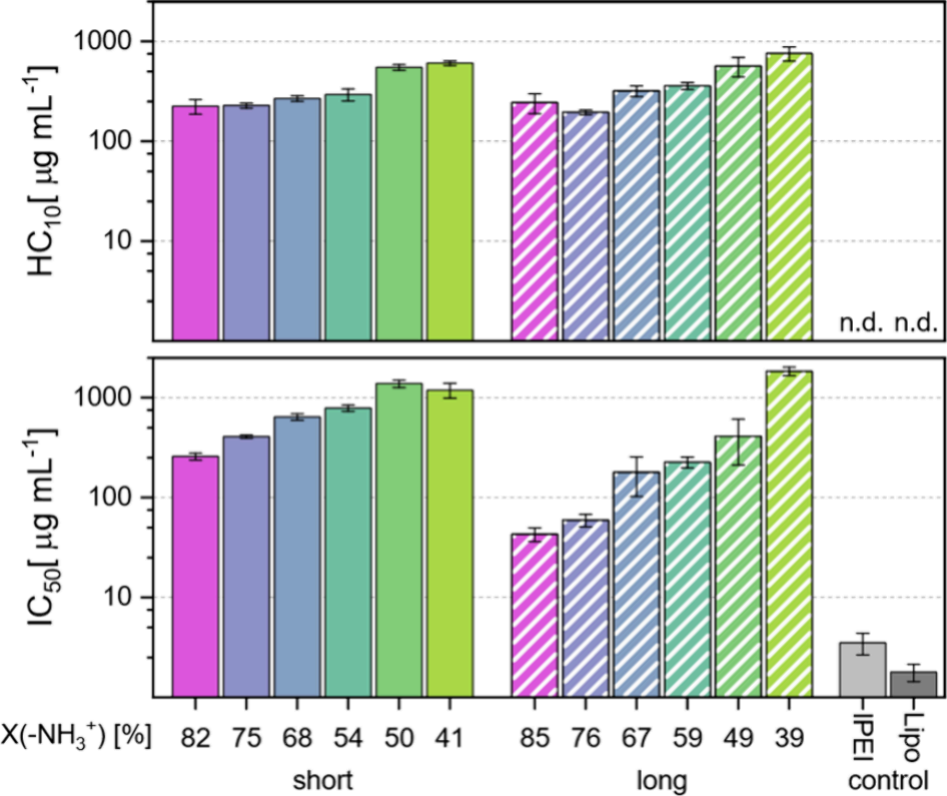
HC_10_ and IC_50_ values of polyvinyl amines determined by hemolysis assay of sheep red blood cells and MTT assay of human MDA-MB-468 breast cancer cells. The transfection agents Lipofectamine 2000 (abbreviated as Lipo), and lPEI (*M*_n_ = 25 kDa) were tested for MDA-MB-468 viability for comparison.

### Transfection of MDA-MB-468 Cells

To assess the ability of the polymers to be used as transfection reagents, a *Renilla* luciferase assay was employed with the pGL4.73 reporter plasmid as a transfection control. The plasmid pGL4.73 was combined with the polymer and added to MDA-MB-468 cells. Transfection of the cells with this reporter plasmid then led to the expression of *Renilla* luciferase. This protein catalyzes the oxidation of coelenterazine, leading to emission of light, which can then be detected and correlated with the degree of transfection that occurred. Lipo and lPEI were again used as references. For initial comparison, the optimal transfection conditions for lPEI (see the ), according to the literature, were chosen for all substances except Lipo, which was used according to the manufacturer’s protocol.^58–60^ The conditions were therefore optimal for the performance of the reference substances PEI and Lipo. The results of the *Renilla* luciferase assay (Figure 4) showed that even under the initial conditions (assumed to be suboptimal) used for PVAms, some perform similarly to PEI. The tested polymers might therefore have achieved better transfection if optimization of the polyplexation conditions was performed before transfection. On the basis of this initial screening, however, compound P75s seems to be the most promising candidate for further optimization.

**Figure 4:**
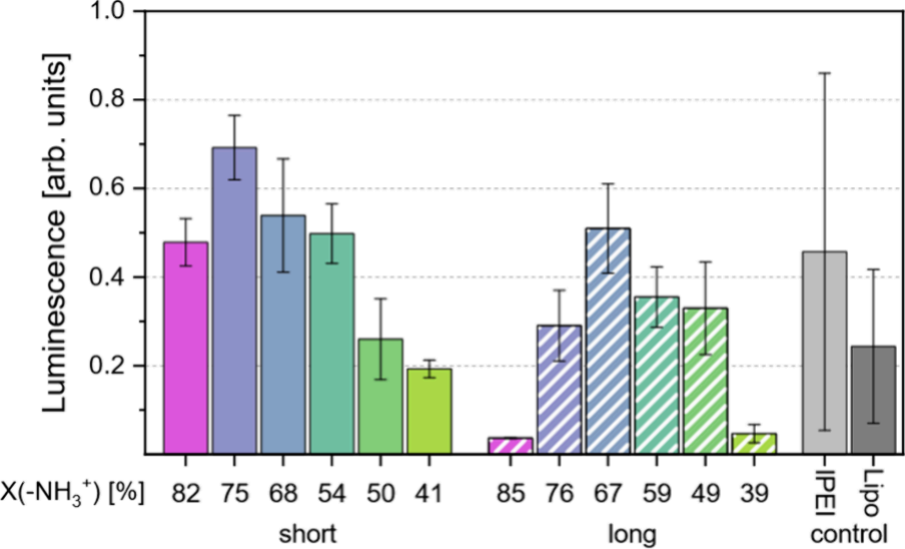
Luminescence intensity measured for the *Renilla* luciferase transfection assay performed on MDA-MB-468 cells using synthesized PVAm copolymers as well as lPEI (25 kDa) and Lipofectamine 2000 (Lipo) as transfection agents.

### Polyplexation Optimization

To this end, lPEI and P75s were both simultaneously optimized to allow for a comparison of these two compounds under conditions allowing for the highest transfection performance. First, the polyplexation capacity of the polymers with plasmid DNA was assessed over a range of N/P ratios using ethidium bromide (EB), a cationic dye that can intercalate DNA strands.^61^ The intercalation of EB leads to an increase in fluorescence. Incubating pGL4.73 with EB and subjecting this complex to lPEI and P75s at different concentrations resulted in a sigmoidal decrease in fluorescence intensity due to the gradual displacement of EB by the polymer (Figure 5). The beginning of the lower plateau represents the lowest N/P ratio at which all of the DNA is bound to the polymer and full condensation of DNA is reached. To obtain comparable values, the points at which 10% (from plateau to plateau) of the increased EB fluorescence remained (IC_10_) were determined via interpolation from the four-parameter logistical fits. These N/P ratios are not necessarily the best ratios for the transfection of cells but instead represent the lowest polymer amount at which the polyplexation process of the DNA reaches 90%. For polyplexation in water, N/P ratios of 2/1 (NP(2)) for P75s and NP(4) for PEI are sufficient to reach the maximum displacement of EB on the basis of their IC_10_ values. It is also evident that an increase in the incubation time does not yield a greater displacement of EB. The polyplexation of DNA by the polymer in pure water is therefore comparably fast. The same experiment with phosphate-buffered saline (PBS) as the buffer medium revealed a significant difference in the fluorescence levels depending on the incubation time. After 4 h, the fluorescence was much lower for both polymers than after 5 min. This finding demonstrates the effect of the buffer composition on polyplex formation. It is likely that the ions in the PBS shield the charges of the DNA and polymer. In turn, the chance for an interaction between one molecule of DNA and one molecule of polymer is decreased, and the whole process of polyplexation is slowed. The difference in the fluorescence levels of the saturation N/P ratios between different incubation times was even more pronounced when Opti-MEM was used as the buffer medium (Figure 5).

**Figure 5:**
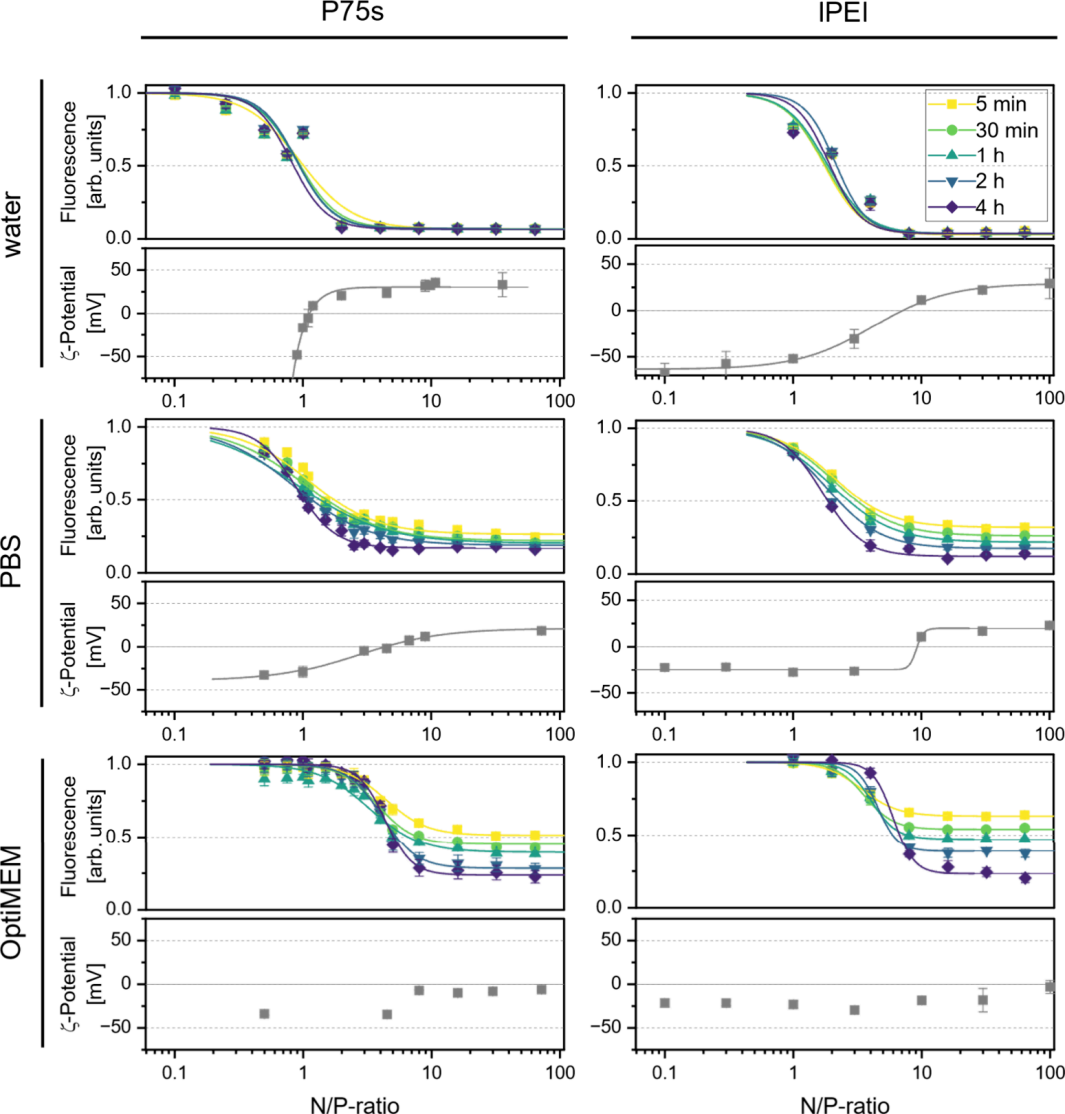
Fluorescence of emission at 590 nm from the ethidium bromide assay and zeta-potential plotted against the N/P ratio of the polymers P75s (left column) and PEI (right column) to pGL4.73 in different buffers: water (top row), PBS (middle row), and OptiMEM (bottom row). Different time intervals of measurement after the initial combination of the polymer-containing solution and the DNA/EtBr-containing solution are plotted in the same corresponding graph. Where applicable, data points were fitted using four-parameter logistical fits.

The increased impact of the medium on polyplexation compared with that of PBS can be attributed to its higher ionic strength and general complexity. This is probably also the cause of a slight shift in the IC_10_ values to slightly higher N/P ratios in Opti-MEM compared to water as medium. The values shift from approximately NP(2) for P75s to NP(7–10) and from NP(4) to NP(7–9) for lPEI (see Table S-EtBr), depending on the incubation time. These results showed that, compared with lPEI, P75s generally needs fewer amine groups per negatively charged phosphate group in DNA to polyplex the plasmid.

To gain more insight into the polyplexation dynamics of the polymers and exclude the possibility that EB interferes with the formation of the polyplexes, zeta potential curves over a range of N/P ratios were measured. The inversion of the surface charge value represents the point at which the polymer has fully screened the nucleic acid charges from the solvent. This value is, in theory, not the same as the IC_10_ value from the ethidium bromide assay, but both values are expected to be similar since both of the underlying states need to be passed on the way to completely polyplex the DNA. These results strengthen this hypothesis since the inversion points are close to the IC_10_ values for PEI and P75s in water and PBS, confirming the results of the ethidium bromide assay and showing that ethidium bromide does not seem to substantially interfere with the formation of the polyplex. For Opti-MEM as a medium, it was unfortunately not possible to measure the point of surface charge inversion due to the abundance of compounds in solution. Since the other two media had a reasonable consensus between the two methods, we considered the IC_10_ determined with the EtBr assay as our basis for the final N/P ratio optimizations. However, higher N/P ratios might still yield better transfection results by assisting in the disruption of the cell membrane.^62^ For this reason, the N/P ratio was also optimized with respect to the transfection ability.

### Transfection Optimization and Comparison

Transfection and impact on cell viability were tested with polyplexes generated by combining the polymers P75s and lPEI with pGL4.7 at N/P ratios starting slightly below the minimal ratio required for full polyplexation as determined above, using a *Renilla* luciferase assay for transfection and a parallel MTT assay for cell viability. OptiMEM was the polyplexation medium of choice because it had the least impact on the cell health of the three media tested previously. As expected,^62^ a slight increase relative to the full polyplexation ratio led to an increase in the transfection efficiency. P75s has a greater transfection efficiency at its NP(16) peak compared with lPEI at the NP(24) peak. However, increasing the N/P ratio too much led to a decrease in the transfection efficiency for both polyplexes (Figure 6). This is most likely due to the toxicity of both polymers beginning to take effect at the NP(12) and higher concentrations. Under the tested conditions, the lPEI-DNA polyplexes appeared to have slightly greater toxicity, which ultimately lowered the viability of the cells to a minimum at NP(64) and NP(96). Accordingly, no significant *Renilla* luciferase activity was detected at these N/P ratios. These results show that P75s is a more versatile transfection reagent for optimizing the N/P ratio. It has a much wider working range of N/P ratios that still allow for decent transfection results as well as a higher maximum possible transfection efficiency.

**Figure 6:**
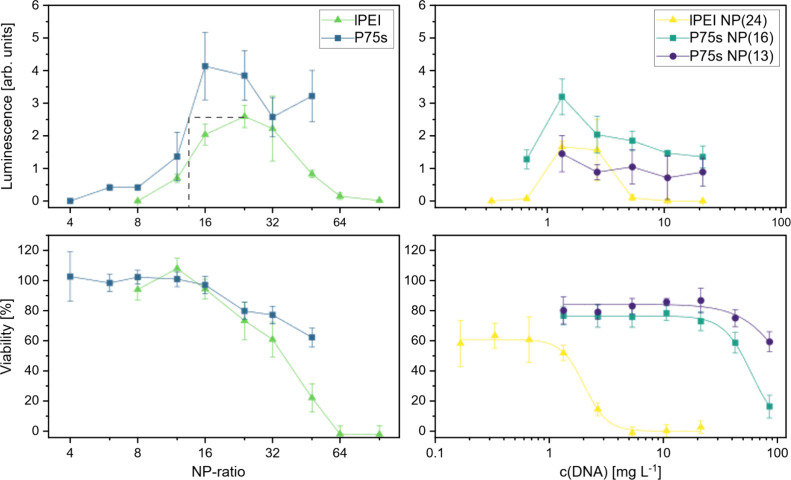
Luminescence intensity measured for the transfection assay of MDA-MB-468 cells with the *Renilla* luciferase plasmid, given in arbitrary units, as well as the percentage of cell viability determined via the MTT assay, for P75s and linear PEI 25 kDa against different N/P ratios at constant c­(DNA) = 1.33 mg/L (left) and at set N/P ratios for P75s and lPEI against the concentration of the polyplex measured as the concentration of DNA used for polyplexation (right). Data points for cell viability under variation of c­(DNA) (bottom right) were fitted applying the Hill equation.

To further investigate the working range of P75s and compare it to that of lPEI, the transfection efficiency and viability of MDA-MB-468 cells exposed to the compounds were analyzed by varying the total concentration of the polyplex and keeping the N/P ratios constant. The selected N/P ratios are those with maximal transfection efficacy for the corresponding compound, which is NP(24) for lPEI and NP(16) for P75s, providing optimal conditions for comparison. P75s was also screened at NP(13), which was estimated to have a transfection efficacy close to the levels of NP(24) for lPEI based on linear interpolation (indicated in Figure 6, top left panel, gray dashed lines). Within the tested concentration range, defined by the amount of pGL4.73 used for polyplex formation, P75s at NP(16) consistently outperformed lPEI at NP(24) at every measured point in terms of the elicited expression of luciferase. Both have peak transfection at 1.3 mg L^–1^, with P75s at NP(16) having an 88% increased signal compared with lPEI at NP(24).

The lPEI NP(24) luciferase signal exhibited a very prominent increase after 0.67 mg L^–1^ and a decrease after 2.7 mg L^–1^, indicating the presence of a sweet spot. P75s at NP(16) comparably increased at 0.67 mg L^–1^ but decreased much more slowly than did lPEI and never reached zero in the given concentration range. P75s at NP(13) has a similar transfection efficiency at 1.3 mg L^–1^ as lPEI at NP(24) but retains the much slower decay of the transfection efficiency of P75s at NP(16). These results show that lPEI has a limited working range and therefore potentially requires more rigorous optimization before a new experiment. P75s, on the other hand, seems to have a much broader working window and enables luciferase expression in the full tested range from 0.33–21 mg L^–1^, even though the peak transfection is reached at 1.3 mg L^–1^, as it is for lPEI. This makes P75s NP(16) an excellent choice for transient transfection and protein expression in research and development, where optimization of transfection and expression conditions is among the most time-consuming factors.

A comparison of NP ratios that exhibit similar transfection rates can also be beneficial in circumstances where a certain level of transfection is needed, but minimizing toxicity or, in general, cell functionality is highly relevant to the experiment.^63^ In terms of the impact of the polyplexes on the viability of MDA-MB-468 cells, lPEI NP(24) had a much stronger adverse effect on cell viability (Figure 6) with an IC_50_ of 2.0 mg L^–1^, whereas P75s had an IC_50_ of 59.3 mg L^–1^. The lPEI NP(24) IC_50_ value is approximately 30 times smaller than the P75s NP(16) IC_50_ value, and the latter generally has better transfection results. P75s NP(13), which has a similar transfection efficacy as lPEI NP(24), has an even higher IC_50,_ which falls outside the range of measured concentrations, and was extrapolated to 124 mg L^–1^. P75s is therefore not only much less toxic than PEI as a pure polymer but also when it is administered as a polyplex. When considering the viability range of PEI and the range of efficient transfection, the sharp decrease in the transfection efficiency between 2.7 mg L^–1^ and 5.3 mg L^–1^ can be linked to its effect on cell viability, indicating strongly that its toxicity is one of the main factors that limits its use, which is in agreement with the prominent literature.^21^ This finding strongly emphasizes the importance of the much lower toxicity of P75s, which truly enables it to serve as a broadly applicable transfection reagent. First results in the mouse fibroblast cell line L-929 also show higher toxicity of the polyplexes of lPEI compared to the ones of P75s (Figure S5), albeit to a lower extend, and strengthen this indication.

The results presented here indicate that P75s should generally be considered an alternative to PEI for transfection purposes. However, its superiority has only been shown in MDA-MB-468 cells and needs to be confirmed in different systems to become the general transfection reagent of choice over PEI.

## Conclusions

In this work, we demonstrated the ability of PI-RAFT polymerization for the synthesis of poly­(NVF-*co*-NVP) with the statistical incorporation of both monomers. NVF repeating units were selectively deprotected under mildly acidic conditions to yield PVAm copolymers. This approach allows control of the charge density in the resulting polycations by varying the comonomer ratio and presents itself as an alternative to routes relying on incomplete hydrolysis. After two sets of polymers with varying charge densities and chain lengths were produced, biocompatibility and gene transfection efficacy were investigated. After identifying P75s as the best performer, we studied the polyplexation process with the aim of identifying the optimal conditions for transfection. We determined that NP(16) and a DNA concentration of 1.33 mg L^–1^ (200 ng/well in a 96-well plate) yielded the best results for the transfection of the tested MDA-MB-468 cell line. Additionally, the gold standard for transfection, PEI,^20,21^ was outperformed significantly in terms of transfection efficiency and, even more so, biocompatibility. For the transfection of MDA-MB-468 cells, P75s should therefore be considered instead of PEI. The general performance as a transfection reagent cannot be inferred from these results alone, but the significant difference in cytotoxicity a significant difference in cytotoxicity was shown in MDA-MB-468 as well as L-929 cells and should translate to different cell lines as well and make an investigation into broader applications worthwhile. Additionally, the implementation of PVAm based structures into advanced polymer drug delivery vehicles is highly incentivized by their high capacity to polyplex nucleic acids of P75s and their relatively high biocompatibility. To facilitate straightforward scale-up, translating the polymer synthesis from batch into flow might also be considered in the future.

## Experimental Section

### Materials

Polyethylenimine, linear, MW 25000 (043896.01), Lipofectamine 2000 (11668027), Leibovitz’s L-15 (11415–049), RPMI 1640 (11875093), heat-inactivated fetal bovine serum (A38400–01), trypsin-EDTA 0.25% (252000–056), Opti-MEM reduced serum medium 1x (31985–047), Dulbecco’s phosphate-buffered saline (DPBS) (14190–144), sodium chloride (207790050), potassium phosphate monobasic (205920025), defibrinated sheep blood Oxoid (12967755) and Immuno Standard Modules Black for 96-well plates (475515) were purchased from Thermo Fisher Scientific (Waltham, MA, USA).

96-well plates for cell culture (83.3924.300) were purchased from Sarstedt (Nümbrecht, Germany). Tissue culture flasks T75/T25 (353136/353108) were purchased from Corning (Corning, NY, USA).

Folded capillary zeta cells (DTS 1070) were purchased from Malvern Panalytical (Malvern, UK). Penicillin/streptomycin (BS. A 2213) was purchased from Bio&SELL GmbH (Nürnberg, Germany). Ethidium bromide (L07482) was purchased from Alfa Aesar (Haverhill, MA, USA). Potassium chloride (60130–1KG) and 3-(4,5-dimethyl-2-thiazolyl)-2,5-diphenyl-2H-tetrazolium bromide (M2128–10G) were purchased from Merck KGaA (Darmstadt, Germany). Sodium phosphate dibasic (1230KG001) was purchased from NeoFroxx (Einhausen, Germany). Dimethyl sulfoxide (AE02.1) and 0.22 μm syringe filters (P666.1) were purchased from Carl Roth (Karlsruhe, Germany). Purified water with a resistance of 18.2 MΩ·cm was drawn from a purelab flex by Veolia (Paris, France). The plasmid pGL4.73 (PR0G0T0S2) was originally from Promega (Walldorf, Germany).

*N*-vinyl formamide (NVF) and *N*-vinyl pyrrolidone (NVP) were purchased from TCI and run through an Al_2_O_3_ (neutral) microcolumn before use to remove the inhibitor. DMSO (99.5%), Et_2_O and acetone (technical grade) were purchased from Carl Roth. Acetone was distilled prior to use. Deuterated solvents for NMR analysis were purchased from Deutero. Xanthate was synthesized according to published protocol.^1^ Deionized water was used for all aqueous systems.

### Instrumentation

NMR spectra were acquired on a Bruker Avance NEO 400 MHz spectrometer using 5 mm diameter tubes and DMSO-*d*_6_ or D_2_O as solvents. The residual signal of the solvent was used for reference, as reported in the literature.^2^

SEC with simultaneous online RI and UV detection was performed on an aqueous system with 0.3% HCOOH and 0.1 M NaCl as additives (pH = 2) with a flow rate of 1 mL min^–1^ at 40 °C. For sample preparation, the polymers were dissolved in up to 1.5 mL of eluent and filtered through 0.45 μm PTFE syringe filters. For each run, 100 μL of sample was injected for each run. For calibration, PEG standards purchased from PSS were used.

For photoinitiation of the polymerizations, a PhotoCube from ThalesNano was used in high mode with all four LED panels being active.

Further details on methods and reaction protocols can be found in the Supporting Information.

## Supplementary Material





## Data Availability

The experimental raw and analyzed data supporting this study can be found in the Supporting Information.

## Notes

Experimental details, additional SEC and NMR data (ZIP)
Figure S1: ^1^H-NMR (400 MHz, D_2_O) of a polymerization mixture containing NVP and NVF at a ratio of 1:1; Figure S2: ^1^H-NMR (400 MHz, D2O) of polymers ([NVF]:[NVP] = 1:1) before and after treatment with HCl and subsequent precipitation in acetone; Figure S3: ^1^H-NMR (400 MHz, D2O) of two batches of polymers obtained by acidic hydrolysis of the precursor material to P75s ([NVF]:[NVP] = 4:1) and precipitation in acetone; Figure S4: Superimposed ^1^H-NMR (400 MHz) spectra measured in DMSO-*d*_6_ (top) and D2O (bottom) of PNVP before (purple trace) and after (green trace) treatment with 1 M HCl at 95 °C for 5 h and precipitation in acetone; Figure S5: Percentage of cell viability of mouse L292 fibroblasts determined via the MTT assay for P75s and linear PEI 25kDa at set N/P ratios against the concentration of the polyplex (measured as the DNA concentration c­(DNA)) used for polyplexation (PDF)

## References

[1] Perrin G. Q., Herzog R. W., Markusic D. M. (2019). Update on Clinical Gene Therapy for
Hemophilia. Blood.

[2] Shahryari A., Saghaeian Jazi M., Mohammadi S., Razavi Nikoo H., Nazari Z., Hosseini E. S., Burtscher I., Mowla S. J., Lickert H. (2019). Development and Clinical
Translation
of Approved Gene Therapy Products for Genetic Disorders. Front Genet.

[3] Sayed N., Allawadhi P., Khurana A., Singh V., Navik U., Pasumarthi S. K., Khurana I., Banothu A. K., Weiskirchen R., Bharani K. K. (2022). Gene Therapy: Comprehensive Overview and Therapeutic
Applications. Life Sciences.

[4] Saeb S., Assche J. V., Loustau T., Rohr O., Wallet C., Schwartz C. (2022). Suicide Gene Therapy
in Cancer and HIV-1 Infection:
An Alternative to Conventional Treatments. Biochem.
Pharmacol..

[5] Chen Y. H., Keiser M. S., Davidson B. L. (2018). Viral Vectors for Gene Transfer. Curr. Protoc Mouse Biol..

[6] Thapa
Magar K., Boafo G. F., Li X., Chen Z., He W. (2022). Liposome-Based Delivery of Biological Drugs. Chin. Chem. Lett..

[7] Kargaard A., Sluijter J. P. G., Klumperman B. (2019). Polymeric
siRNA Gene Delivery - Transfection
Efficiency versus Cytotoxicity. J. Controlled
Release.

[8] Cai X., Dou R., Guo C., Tang J., Li X., Chen J., Zhang J. (2023). Cationic Polymers
as Transfection Reagents for Nucleic Acid Delivery. Pharmaceutics.

[9] Li S., Huang L. (2000). Nonviral Gene Therapy: Promises and Challenges. Gene Ther..

[10] White J. M., Whittaker G. R. (2016). Fusion of Enveloped Viruses in Endosomes. Traffic.

[11] Baden L. R., El Sahly H. M., Essink B., Kotloff K., Frey S., Novak R., Diemert D., Spector S. A., Rouphael N., Creech C. B., McGettigan J., Khetan S., Segall N., Solis J., Brosz A., Fierro C., Schwartz H., Neuzil K., Corey L., Gilbert P., Janes H., Follmann D., Marovich M., Mascola J., Polakowski L., Ledgerwood J., Graham B. S., Bennett H., Pajon R., Knightly C., Leav B., Deng W., Zhou H., Han S., Ivarsson M., Miller J., Zaks T. (2021). Efficacy and Safety
of the mRNA-1273 SARS-CoV-2 Vaccine. N. Engl.
J. Med..

[12] Polack F. P., Thomas S. J., Kitchin N., Absalon J., Gurtman A., Lockhart S., Perez J. L., Pérez Marc G., Moreira E. D., Zerbini C., Bailey R., Swanson K. A., Roychoudhury S., Koury K., Li P., Kalina W. V., Cooper D., Frenck R. W., Hammitt L. L., Türeci Ö., Nell H., Schaefer A., Ünal S., Tresnan D. B., Mather S., Dormitzer P. R., Şahin U., Jansen K. U., Gruber W. C. (2020). Safety and Efficacy
of the BNT162b2 mRNA Covid-19 Vaccine. N. Engl.
J. Med..

[13] Hou X., Zaks T., Langer R., Dong Y. (2021). Lipid Nanoparticles
for mRNA Delivery. Nat. Rev. Mater..

[14] Wang J., Ding Y., Chong K., Cui M., Cao Z., Tang C., Tian Z., Hu Y., Zhao Y., Jiang S. (2024). Recent Advances in Lipid Nanoparticles and Their Safety Concerns
for mRNA Delivery. Vaccines.

[15] Javed S., Mangla B., Almoshari Y., Sultan M. H., Ahsan W. (2022). Nanostructured
Lipid Carrier System: A Compendium of Their Formulation Development
Approaches, Optimization Strategies by Quality by Design, and Recent
Applications in Drug Delivery. Nanotechnol.
Rev..

[16] Monnery B. D., Wright M., Cavill R., Hoogenboom R., Shaunak S., Steinke J. H. G., Thanou M. (2017). Cytotoxicity of Polycations:
Relationship of Molecular Weight and the Hydrolytic Theory of the
Mechanism of Toxicity. Int. J. Pharm..

[17] Haladjova E., Halacheva S., Posheva V., Peycheva E., Moskova-Doumanova V., Topouzova-Hristova T., Doumanov J., Rangelov S. (2015). Comblike Polyethylenimine-Based
Polyplexes: Balancing Toxicity, Cell Internalization, and Transfection
Efficiency via Polymer Chain Topology. Langmuir.

[18] Floyd T. G., Song J.-I., Hapeshi A., Laroque S., Hartlieb M., Perrier S. (2022). Bottlebrush Copolymers for Gene Delivery: Influence
of Architecture, Charge Density, and Backbone Length on Transfection
Efficiency. J. Mater. Chem. B.

[19] Wang C., He W., Wang F., Yong H., Bo T., Yao D., Zhao Y., Pan C., Cao Q., Zhang S., Li M. (2024). Recent Progress of
Non-Linear Topological Structure Polymers: Synthesis,
and Gene Delivery. J. Nanobiotechnol.

[20] Bus T., Traeger A., Schubert U. S. (2018). The Great
Escape: How Cationic Polyplexes
Overcome the Endosomal Barrier. J. Mater. Chem.
B.

[21] Casper J., Schenk S. H., Parhizkar E., Detampel P., Dehshahri A., Huwyler J. (2023). Polyethylenimine (PEI) in Gene Therapy: Current Status
and Clinical Applications. J. Controlled Release.

[22] Fattahi N., Gorgannezhad L., Masoule S. F., Babanejad N., Ramazani A., Raoufi M., Sharifikolouei E., Foroumadi A., Khoobi M. (2024). PEI-Based Functional
Materials: Fabrication
Techniques, Properties, and Biomedical Applications. Adv. Colloid Interface Sci..

[23] Jäger M., Schubert S., Ochrimenko S., Fischer D., Schubert U. S. (2012). Branched
and Linear Poly­(Ethylene Imine)-Based Conjugates: Synthetic Modification,
Characterization, and Application. Chem. Soc.
Rev..

[24] Tian Y., Zhao Y., Yin C., Tan S., Wang X., Yang C., Zhang T.-D., Zhang X., Ye F., Xu J., Wu X., Ding L., Zhang J., Pei J., Wang X.-T., Zhang R. X., Xu J., Wang W., Filipe C. D. M., Hoare T., Yin D.-C., Qian A., Deng X. (2022). Polyvinylamine with Moderate Binding Affinity as a Highly Effective
Vehicle for RNA Delivery. J. Controlled Release.

[25] Dréan M., Debuigne A., Goncalves C., Jérôme C., Midoux P., Rieger J., Guégan P. (2017). Use of Primary
and Secondary Polyvinylamines for Efficient Gene Transfection. Biomacromolecules.

[26] Dréan M., Debuigne A., Jérôme C., Goncalves C., Midoux P., Rieger J., Guégan P. (2018). Poly­(N-Methylvinylamine)-Based
Copolymers for Improved Gene Transfection. Macromol.
Biosci.

[27] Vinogradoff V., Duvernay F., Farabet M., Danger G., Theulé P., Borget F., Guillemin J. C., Chiavassa T. (2012). Acetaldehyde
Solid State Reactivity at Low Temperature: Formation of the Acetaldehyde
Ammonia Trimer. J. Phys. Chem. A.

[28] Kanto R., Yonenuma R., Yamamoto M., Furusawa H., Yano S., Haruki M., Mori H. (2021). Mixed Polyplex
Micelles with Thermoresponsive
and Lysine-Based Zwitterionic Shells Derived from Two Poly­(Vinyl Amine)-Based
Block Copolymers. Langmuir.

[29] Maki Y., Mori H., Endo T. (2007). Controlled
RAFT Polymerization of
N -Vinylphthalimide and Its Hydrazinolysis to Poly­(Vinyl Amine). Macro Chemistry & Physics.

[30] Achari A. E., Coqueret X., Lablache-Combier A., Loucheux C. (1993). Preparation of Polyvinylamine
from Polyacrylamide: A Reinvestigation of the Hofmann Reaction. Makromol. Chem..

[31] An Z., Cao B., Zhang J., Zhang B., Zhou C., Hu X., Chen W. (2022). Efficient
Transient Expression of Plasmid DNA Using Poly (2-(N,N-Dimethylamino)
Ethyl Methacrylate) in Plant Cells. Front Bioeng
Biotechnol.

[32] Pinschmidt R. K. (2010). Polyvinylamine
at Last. J. Polym. Sci. A Polym. Chem..

[33] Yamamoto K., Imamura Y., Nagatomo E., Serizawa T., Muraoka Y., Akashi M. (2003). Synthesis and Functionalities
of Poly­(N -vinylalkylamide).
XIV. Polyvinylamine Produced by Hydrolysis of Poly­(N -vinylformamide)
and Its Functionalization. J. Appl. Polym. Sci..

[34] Gu L., Zhu S., Hrymak A. N. (2002). Acidic
and Basic Hydrolysis of Poly­(N-vinylformamide). J. Appl. Polym. Sci..

[35] Nakabayashi K., Mori H. (2013). Recent Progress in
Controlled Radical Polymerization of N-Vinyl Monomers. Eur. Polym. J..

[36] Dréan M., Guégan P., Detrembleur C., Jérôme C., Rieger J., Debuigne A. (2016). Controlled Synthesis of Poly­(Vinylamine)-Based
Copolymers by Organometallic-Mediated Radical Polymerization. Macromolecules.

[37] Dupre--Demorsy A., Coutelier O., Destarac M., Nadal C., Bourdon V., Ando T., Ajiro H. (2022). RAFT Polymerization of N-Methyl-N-Vinylacetamide
and Related Double Hydrophilic Block Copolymers. Macromolecules.

[38] Dupre--Demorsy A., Kurowska I., Balayssac S., Hennetier M., Ric A., Bourdon V., Ando T., Ajiro H., Coutelier O., Destarac M. (2022). RAFT Polymerisation
of N -Vinylformamide and the Corresponding
Double Hydrophilic Block Copolymers. Polym.
Chem..

[39] Stenzel M. H., Barner-Kowollik C. (2016). The Living Dead – Common Misconceptions about
Reversible Deactivation Radical Polymerization. Mater. Horiz..

[40] Hartlieb M. (2022). Photo-Iniferter
RAFT Polymerization. Macromol. Rapid Commun..

[41] Lehnen A.-C., Kurki J. A. M., Hartlieb M. (2022). The Difference between Photo-Iniferter
and Conventional RAFT Polymerization: High Livingness Enables the
Straightforward Synthesis of Multiblock Copolymers. Polym. Chem..

[42] Perrier S. (2017). 50th Anniversary
Perspective: RAFT Polymerization–A User Guide. Macromolecules.

[43] Beres M. A., Boyer C., Hartlieb M., Konkolewicz D., Qiao G. G., Sumerlin B. S., Perrier S. (2025). RAFT with
Light: A
User Guide to Using Thiocarbonylthio Compounds in Photopolymerizations. ACS Polym. Au.

[44] Kurowska I., Dupre-Demorsy A., Balayssac S., Hennetier M., Ric A., Bourdon V., Ando T., Ajiro H., Coutelier O., Destarac M. (2023). Tailor-Made Poly­(Vinylamine) via Purple LED-Activated
RAFT Polymerization of N-Vinylformamide. Macromol.
Rapid Commun..

[45] Wang H., Li Q., Dai J., Du F., Zheng H., Bai R. (2013). Real-Time
and in Situ Investigation of “Living”/Controlled Photopolymerization
in the Presence of a Trithiocarbonate. Macromolecules.

[46] Cho J.-H., Ko J.-Y., Shin J.-M., Kwark Y.-J. (2025). Enhancing Control
in the Raft Polymerization of N-Vinyl Formamide Through Low-Temperature
Photoinitiated Copolymerization with N-Vinyl Pyrrolidone. Social Science Research Network: Rochester, NY.

[47] Pinschmidt R. K., Wasowski L. A., Orphanides G. G., Yacoub K. (1996). Amine Functional Polymers
Based on *N*-Ethenylformamide. Prog. Org. Coat..

[48] Gu L., Zhu S., Hrymak A. N. (2002). Acidic and Basic Hydrolysis of Poly­(N-vinylformamide). J. Appl. Polym. Sci..

[49] Khondee S., Yakovleva T., Berkland C. (2010). Low Charge Polyvinylamine Nanogels
Offer Sustained, Low-level Gene Expression. J. Appl. Polym. Sci..

[50] Cailleau R., Olivé M., Cruciger Q. V. (1978). Long-Term Human Breast Carcinoma
Cell Lines of Metastatic Origin: Preliminary Characterization. In Vitro.

[51] Vieira P., Jesus V., Cândido M. A., Pacheco-Soares C., Castilho M., Raniero L. (2022). Specific Nanomarkers
Fluorescence:
In Vitro Analysis for EGFR Overexpressed Cells in Triple-Negative
Breast Cancer and Malignant Glioblastoma. Photodiagnosis
and Photodynamic Therapy.

[52] Burness M. L., Grushko T. A., Olopade O. I. (2010). Epidermal Growth
Factor Receptor
in Triple-Negative and Basal-Like Breast Cancer: Promising Clinical
Target or Only a Marker?. Cancer Journal.

[53] Mustafa E. H., Laven-Law G., Kikhtyak Z., Nguyen V., Ali S., Pace A. A., Iggo R., Kebede A., Noll B., Wang S., Winter J. M., Dwyer A. R., Tilley W. D., Hickey T. E. (2024). Selective
Inhibition of CDK9 in Triple Negative Breast
Cancer. Oncogene.

[54] Won E.-J., Lee M., Lee E.-K., Baek S.-H., Yoon T.-J. (2024). Lipid-Based Nanoparticles
Fused with Natural Killer Cell Plasma Membrane Proteins for Triple-Negative
Breast Cancer Therapy. Pharmaceutics.

[55] Wang T., Larcher L. M., Ma L., Veedu R. N. (2018). Systematic Screening
of Commonly Used Commercial Transfection Reagents towards Efficient
Transfection of Single-Stranded Oligonucleotides. Molecules.

[56] Kwolek U., Jamróz D., Janiczek M., Nowakowska M., Wydro P., Kepczynski M. (2016). Interactions of Polyethylenimines
with Zwitterionic and Anionic Lipid Membranes. Langmuir.

[57] Fischer D., Li Y., Ahlemeyer B., Krieglstein J., Kissel T. (2003). In Vitro Cytotoxicity
Testing of Polycations: Influence of Polymer Structure on Cell Viability
and Hemolysis. Biomaterials.

[58] Ong C. E. B., Patchett A. L., Darby J. M., Chen J., Liu G.-S., Lyons A. B., Woods G. M., Flies A. S. (2021). NLRC5 Regulates
Expression of MHC-I and Provides a Target for Anti-Tumor Immunity
in Transmissible Cancers. J. Cancer Res. Clin
Oncol.

[59] Longo P. A., Kavran J. M., Kim M.-S., Leahy D. J. (2013). Transient
Mammalian
Cell Transfection with Polyethylenimine (PEI). Methods Enzymol..

[60] de
los Milagros Bassani Molinas M., Beer C., Hesse F., Wirth M., Wagner R. (2014). Optimizing the Transient Transfection
Process of HEK-293 Suspension Cells for Protein Production by Nucleotide
Ratio Monitoring. Cytotechnology.

[61] Geall A. J., Blagbrough I. S. (2000). Rapid and
Sensitive Ethidium Bromide Fluorescence Quenching
Assay of Polyamine Conjugate–DNA Interactions for the Analysis
of Lipoplex Formation in Gene Therapy. J. Pharm.
Biomed. Anal..

[62] Monnery B. D. (2021). Polycation-Mediated
Transfection: Mechanisms of Internalization and Intracellular Trafficking. Biomacromolecules.

[63] Schakowski F., Buttgereit P., Mazur M., Märten A., Schöttker B., Gorschlüter M., Schmidt-Wolf I. G. (2004). Novel Non-Viral
Method for Transfection of Primary Leukemia Cells and Cell Lines. Genet Vaccines Ther.

