# Supplementary material for: Copolymers of NVAm and NVP for Efficient Gene Delivery
Source: ACS Polym Au. 2026 Jan 16;6(1):426–36. doi: 10.1021/acspolymersau.5c00167 (PMC12903468; doi:10.1021/acspolymersau.5c00167)
Supplement: Supplementary file 2 [file lg5c00167_si_002.pdf]

Supporting Information

Copolymers of NVAm and NVP for efficient gene delivery

Tom Fielitz,[a,+] Christopher Raab,[b,d,+] Vitalii Tkachenko,[a] Kristine M. Oleszkiewicz,[b,e] Hendrik Fuchs,[b,*] Matthias Hartlieb[a,c,*]

a – Institute of Chemistry, University of Potsdam, Karl-Liebknecht-Straße 24-25, DE-14476 Potsdam, Germany.

b – Charité – Universitätsmedizin Berlin, corporate member of Freie Universität Berlin and Humboldt-Universität zu Berlin, Institute of Diagnostic Laboratory Medicine, Clinical Chemistry and Pathobiochemistry, Augustenburger Platz 1, DE-13353 Berlin.

c – Fraunhofer Institute for Applied Polymer Research IAP, Geiselbergstraße 69, 14476 Potsdam.

d – Institute of Pharmacy, Freie Universität Berlin, Königin-Luise-Straße 2 and 4, DE-14195 Berlin.

e – Institute of Chemistry and Biochemistry, Freie Universität Berlin, Arnimallee 20, DE-14195 Berlin.

+ – These authors contributed equally to this work.

* Correspondence to hendrik.fuchs@charite.de and mhartlieb@uni-potsdam.de

Materials & Instrumentation

Information on Materials and Instrumentation can be found in the main manuscript.

Methods

General procedure for PI-RAFT polymerizations

Monomers were prepared in glass reaction vials (5 mL test tubes, Pyrex) after removal of the inhibitors. An appropriate amount of freshly prepared xanthate stock solution in the reaction solvent was added, and the mixture was topped with solvent to reach the desired volume. After an aliquant was removed for NMR analysis, the reaction vessels were capped with rubber septa and bubbled with N_2 for 15 min to replace O_2 before being placed in the reactor chamber of the PhotoCube. The reactions were performed without stirring. For purification, the mixtures were precipitated in acetone and redissolved in water three times before being precipitated one final time, and the residual solvent was removed under reduced pressure. When stored in the dark at 4 °C, polymers are stable for at least 1.5 years.

Kinetic analyses

Polymerization mixtures were prepared as described above, using DMSO-d_6 as the solvent. The photoreactor was turned off at regular intervals, and aliquots (150 µL) were taken with N_2-flushed syringes. A 50 µL sample was diluted in 600 µL D_2O and subjected to ^{1}H-NMR analysis.

Determination of monomer conversion

Monomer conversion was determined from ^{1}H-NMR analysis. To determine the relative concentration of residual monomers, the intensity of signals related to the vinyl protons was compared to the signal of the formyl proton in *N*-vinyl formamide (NVF) (Figure S1). To determine *N*-vinyl pyrrolidone (NVP) conversion during copolymerizations, the intensity of the latter was multiplied with the initial ratio of NVF to NVP.

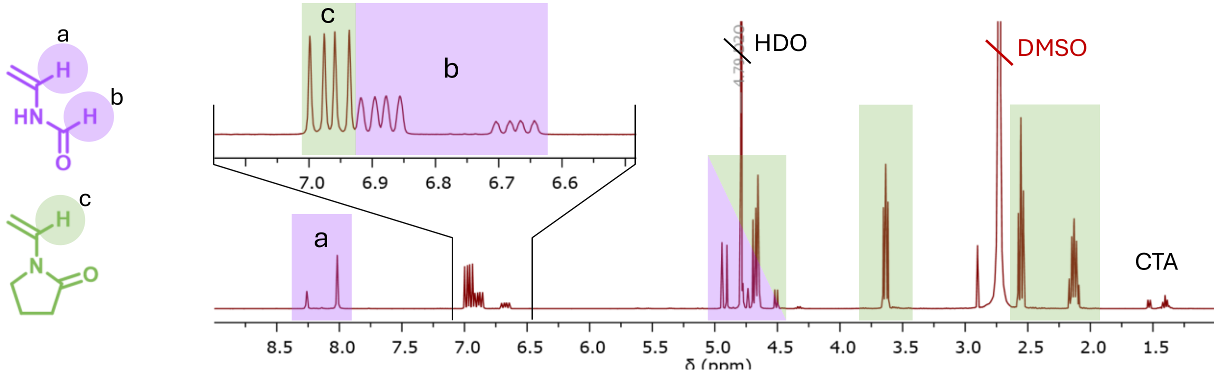

Figure S1: ^{1}H-NMR (400 MHz, D_2O) of a polymerization mixture containing NVP and NVF at a ratio of 1:1. The peaks used for determination of conversion are labeled. The apparent dissolution of the signal corresponding to proton **b** is explained by the presence of the cis- and trans-isomers of NVF.[3]

Acidic hydrolysis of polymers

In a 10-mL test tube with glass fitting, the polymer was dissolved in 1 M HCl at a concentration of 25 mg mL^{-1}. The tube was closed tightly with a glass stopper and placed in an oil bath at 95 °C. The amount of residual formamide was determined via comparison of the intensity of the formyl proton signal in the ^{1}H-NMR with that of the polymer backbone (Figure S2). Polymers were stored in the dark at 4 °C and remain stable for at least 1.5 years.

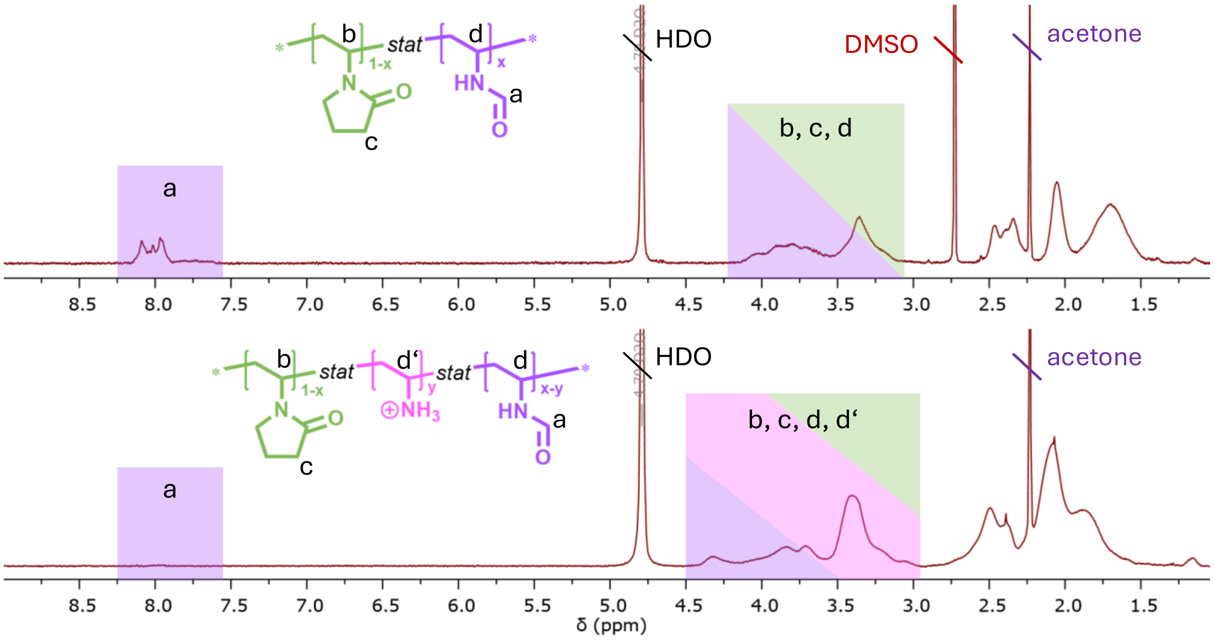

Figure S2: ^{1}H-NMR (400 MHz, D_2O) of polymers ([NVF]:[NVP] = 1:1) before (top) and after (bottom) treatment with HCl and subsequent precipitation in acetone. Areas used for quantification of residual formamide are marked.

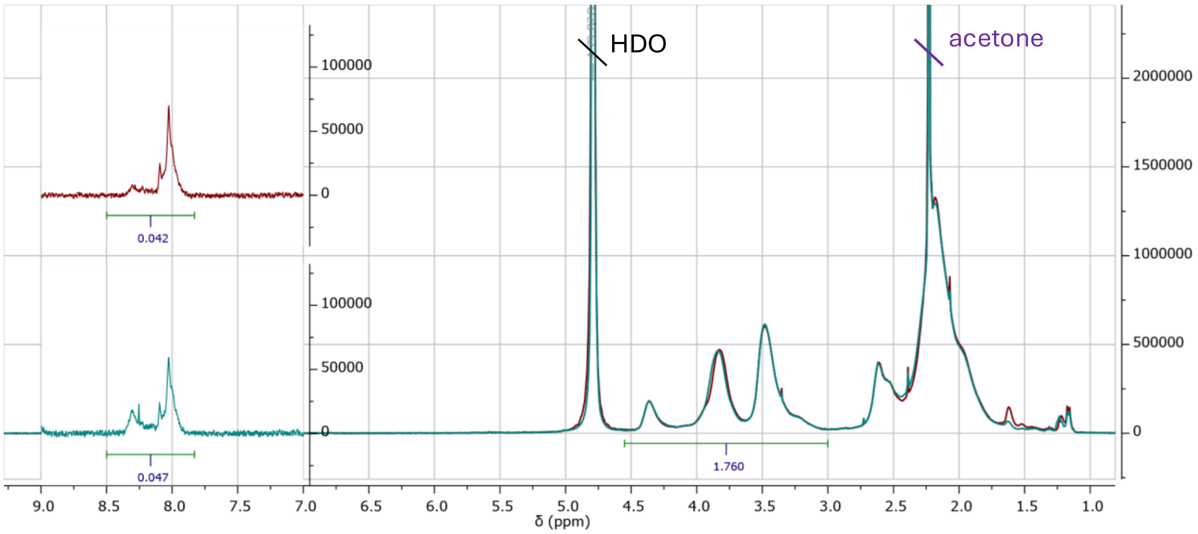

Figure S3: ^{1}H-NMR (400 MHz, D_2O) of two batches of polymers obtained by acidic hydrolysis of the precursor material to P75s ([NVF]:[NVP] = 4:1) and precipitation in acetone. The area 7.0 – 9.0 has been zoomed in to allow comparison in the residual formyl proton signal.

Synthesis of poly(N-vinyl pyrrolidone) via free radical polymerization

To 222 mg of *N*-vinyl pyrrolidone (NVP, 2 mmol) in a 5 mL test tube (Pyrex), a solution of AIBN (1.6 mg, 0.1 mmol) in 780 mg of tetrahydrofuran (THF) was added. To allow monitoring of monomer conversion via ^{1}H-NMR, toluene (20 µL) was added as internal standard. The mixture was homogenized and sparged with nitrogen for 10 minutes. Afterwards, an aliquant (40 µL) was taken using a nitrogen flushed syringe and dissolved in 600 µL of DMSO-d_6 for NMR analysis. The mixture was placed into an oil bath preheated to 60 °C and quenched after 16.5 h by cooling to 0 °C and sparging with air. Again, an aliquant was taken and dissolved in 600 µL of DMSO-d_6 for NMR analysis. Monomer conversion was determined to be 97% by comparison of the relative signal intensity of the NVP vinyl proton (δ = 7.03 - 6.885 ppm) to the signal of the aromatic protons of the internal toluene standard (δ = 7.3 - 7.10 ppm). For purification, the polymer was precipitated in Et_2O; the white precipitate redissolved in DMSO and again precipitated in Et_2O, before being washed with Et_2O two times. The white precipitate was dried at room temperature under reduced pressure, yielding 185 mg of white powder.

Hydrolysis trial of poly(NVP)

PNVP (50 mg) was dissolved in 1 M HCl (2 mL) in an 8 mL test tube. The vial was closed with a glass stopper and placed into a preheated oil bath at 95 °C. After 5 h, the polymer was purified by precipitation in acetone and subsequent washing. The precipitate was dried at under reduced pressure yielding 45 mg of white powder.

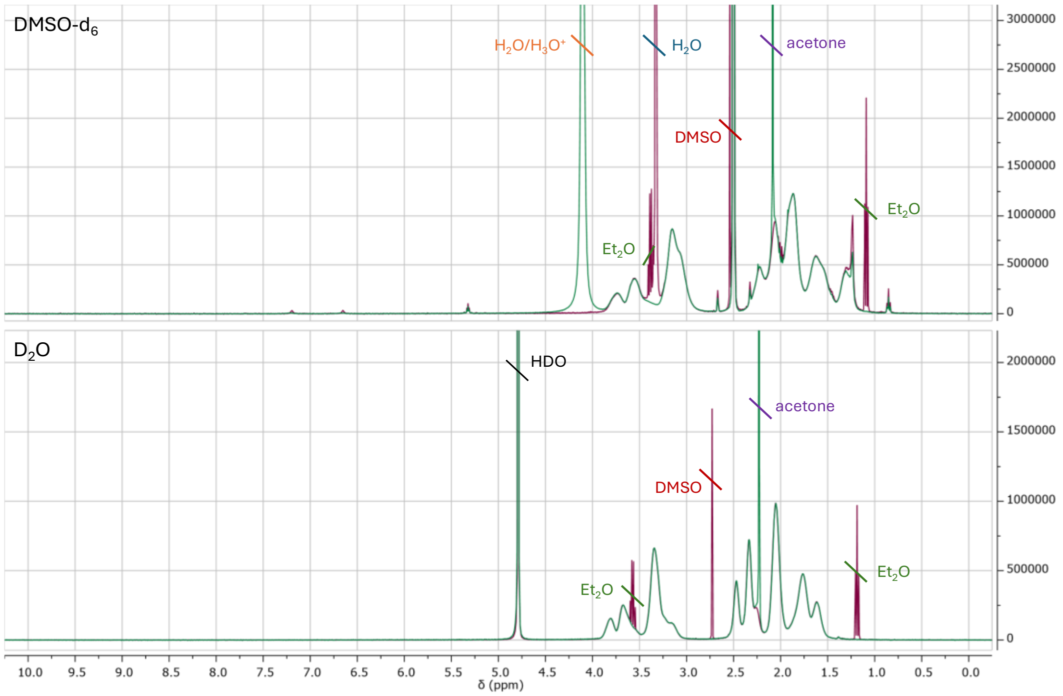

Figure S4: Superimposed ^{1}H-NMR (400 MHz) spectra measured in DMSO-d_6 (top) and D_2O (bottom) of PNVP before (purple trace) and after (green trace) treatment with 1 M HCl at 95 °C for 5 h and precipitation in acetone. Spectra appear identical apart from residual signal of solvents used for NMR (HDO, DMSO-d_5), or in precipitation (Et_2O, DMSO, acetone). The apparent shift of the signal corresponding to H2O in DMSO indicates the presence of residual HCl in the mixture, as the protons of H_3O^+ are much more deshielded, therefore appearing further downfield. Due to the fast exchange of H^+, signals for H_3O^+ and H_2O are merged.

Potentiometric titration

A general-purpose pH probe connected to a Mettler Toledo pH meter was used for potentiometric titration. The pH meter was rinsed with deionized water and calibrated with standard buffers (pH 4, pH 7, and pH 10). All solutions were prepared with Milli-Q water and titrations were carried out at ambient temperature (25 °C) and under gentle mixing with a magnetic stirrer. The titrate solution (3 mL) prepared at a concentration of 1 mg mL^{-1} of polymers in HCl (0.01 M) was placed in the titration cell (10 ml scintillation vial). NaOH (0.1 M) was gradually added as titrant with Eppendorf micropipettes (from 20 µL to 600 µL). Sufficient time (approximately 1 min) was necessary to reach a stable pH reading before the next addition of the base. The recorded pH values were plotted against the respective cumulative volumes of the titrant to obtain the titration curve and the first derivatives.

Hemocompatibility tests

The hemocompatibility of the polymers was determined via defibrinated sheep blood erythrocytes following an established procedure reported previously.[4] The interaction with the polymers was probed via a dilution series starting at a concentration of 12 µg mL^{-1} and diluting at a 1:1 ratio for 9 steps up to 6144 µg mL^{-1}.

Ethidium Bromide Replacement Assay

Stock solutions of pGL4.73 and ethidium bromide in water were diluted in the respective buffers (e.g., phosphate-buffered saline) to yield solutions with concentrations of 4.44 mg L^{-1} pGL4.73 and 1.11 mg L^{-1} EtBr. This solution was kept from light whenever possible. The polymer in question was diluted to a concentration ten times the concentration of interest in the buffer solution, and 10 µL was added to a well of a black 96-well plate. The DNA/ethidium bromide mixture (90 µL) was added to all the wells containing the samples. The plate was covered with aluminum foil and shaken at medium speed at room temperature while being incubated. All the wells in the plate were measured via a SPECTRA MAX GEMINI (Molecular Devices, San José, USA, CA). Excitation was set to 260 nm, and detection was set to 590 nm. Negative controls were measured without any polymer. The background was measured by the use of samples without DNA. The samples were measured at 5 and 30 min, as well as at 1, 2 and 4 h.

Zeta Potential

Stock solutions of the polymers and DNA in the buffer of interest were prepared at double the desired concentration before combining them in equal parts to reach the final concentration. The samples were vortexed briefly and incubated for 10 min at room temperature. The samples were then transferred to a DTS 1070 cuvette and inserted into a Zetasizer (ULTRA). The zeta potential was measured in triplicate in auto mode at 25 °C after 2 min of equilibration. For the different buffers (water, PBS, and OptiMEM), the refractive index (1.33, 1.33, and 1.34) and viscosity (0.8872, 0.8882, and 1.1) parameters varied slightly.

Cell Culturing

MDA-MB-468 cells were cultured in Leibovitz's L-15 medium supplemented with 10% FBS and 1% streptomycin and penicillin at 37 °C (L-15 complete medium) without atmospheric CO_2. Subculturing was performed biweekly by washing the cells with prewarmed PBS (5 mL for T25 flasks and 10 mL for T75 flasks) and then incubating the cells with 0.25% trypsin/EDTA (1 mL for T25 flasks and 2 mL for T75 flasks) for 5–10 minutes until most of the cells were detached. L-15 medium was added (4 mL for the T25 flasks and 8 mL for the T75 flasks), and the suspension was pipetted up and down 3 times. The suspension was transferred to a 15-mL Falcon tube, and the cells were pelleted via centrifugation at 500 × g for 5 min at room temperature. The supernatant was removed, and the cell pellet was resuspended in 1 mL of L-15 complete medium. The suspension was diluted, commonly 1:5, and added to a new tissue culture flask to reach confluency within the desired time range.

L-929 cells were cultured in RPMI 1640 medium supplemented with 10% FBS and 1% streptomycin and penicillin at 37 °C (L-15 complete medium) with 5% CO_2. Subculturing was performed biweekly by washing the cells with prewarmed PBS (5 mL for T25 flasks and 10 mL for T75 flasks) and then incubating the cells with 0.25% trypsin/EDTA (1 mL for T25 flasks and 2 mL for T75 flasks) for 5–10 minutes until most of the cells were detached. L-15 medium was added (4 mL for the T25 flasks and 8 mL for the T75 flasks), and the suspension was pipetted up and down 3 times. The suspension was transferred to a 15-mL Falcon tube, and the cells

were pelleted via centrifugation at 500 × g for 5 min at room temperature. The supernatant was removed, and the cell pellet was resuspended in 1 mL of L-15 complete medium. The suspension was diluted, commonly 1:5, and added to a new tissue culture flask to reach confluency within the desired time range.

Cell Viability Assay with MDA-MB-468 cells

The MDA-MB-468 cells were split and distributed into the wells of a clear 96-well plate at a density of 2,500 cells per well in 100 µL of growth medium. After 24 h, different concentrations of polymer or polyplex were added to the wells in 50 µL of Opti-MEM. The plates were carefully agitated to allow for sufficient mixing and then incubated for 72 h at 37 °C without atmospheric CO_2. A solution of 5 mg mL^{-1} 3-(4,5-dimethylthiazol-2-yl)-2,5-diphenyltetrazolium bromide (MTT) in ultrapure water was then prepared and filtered through a 0.22 µm syringe filter unit. Subsequently, 30 µL of the solution was added to each well and agitated to allow mixing. The plate was incubated for an additional 2 h. After this incubation period, the solution was removed, leaving solid formazan crystals at the bottom of the wells. The crystals were fully dissolved in 100 µL of dimethyl sulfoxide (DMSO) by shaking for 5 min on a plate shaker at medium speed. The absorbance of the solution at 570 nm was measured with a SpectraMax 340PC 384 (Molecular Devices, San José, USA, CA).

Transfection Assay

To assess the transfection efficiency of each polymer, the Luc-Pair™ Renilla Luciferase HS Assay Kit (LF011) from GeneCopoeia (Rockville, MD, USA) was used according to the manufacturer’s protocol. In brief, MDA-MB-468 cells were cultured in 100 µL of Leibovitz's L-15 medium at 37 °C without atmospheric CO_2 in 96-well plates until a confluency of approximately 50% was reached. The cells were then transfected with 200 ng of the pGL4.73 plasmid and the transfection reagent in an amount corresponding to the required N/P ratio by diluting the needed volume to 25 µL in OptiMEM, incubating it at room temperature for 5 min and combining it before being incubated for another 15 min at room temperature to finally add the mixture to the cells. After 48 h, the culture medium was removed from the wells, and the cells were washed with 200 µL of PBS at 4 °C. The buffer was removed, and 20 µL of RLuc lysis buffer was added to the wells. The plate was shaken for 15 min at medium speed on a VIBRAMAX 100 (Heidolph, Schwabach, Germany). The lysate was transferred to a black 96-well plate. The substrate was prepared according to the protocol of the manufacturer, and 100 µL was injected into the well via the built-in autoinjector of the Fluoroskan Ascent FL (Thermo Scientific, Waltham, MA, USA). The plate was shaken at medium speed and strength for 10 s immediately after each injection and directly measured for 2 s using the chemiluminescence setting. The lysate from untransfected cells served as a negative control, and either samples transfected via linear PEI 25 kDa or Lipofectamine 2000 were used as positive controls. Preliminary tests of the prototypes were conducted with NP(23), as it is often used in reference literature. The ratio is often 3/1 (w/w), which translates to roughly NP(23) by dividing the molar mass of the phosphorus-containing subunit of DNA

(average 330 g mol^{-1}) by the molar mass of the nitrogen-containing subunit of lPEI (43 g mol^{-1}) and multiplying by three for the mass ratios used.[5,6]

Viability Assay of mouse L292 fibroblasts against polyplexes

The L-929 cells were split and distributed into the wells of a clear 96-well plate at a density of 1,250 cells per well in 100 µL of growth medium. After 24 h, different concentrations of polyplex at a set N/P ratio were added to the wells in 50 µL of Opti-MEM. The plates were carefully agitated to allow for sufficient mixing and then incubated for 72 h at 37 °C with supplementation of 5% CO_2. A solution of 5 mg mL^{-1} 3-(4,5-dimethylthiazol-2-yl)-2,5-diphenyltetrazolium bromide (MTT) in ultrapure water was then prepared and filtered through a 0.22 µm syringe filter unit. Subsequently, 30 µL of the solution was added to each well and agitated to allow mixing. The plate was incubated for an additional 2 h. After this incubation period, the solution was removed, leaving solid formazan crystals at the bottom of the wells. The crystals were fully dissolved in 100 µL of dimethyl sulfoxide (DMSO) by shaking for 5 min on a plate shaker at medium speed. The absorbance of the solution at 570 nm was measured with a SpectraMax 340PC 384 (Molecular Devices, San José, USA, CA).

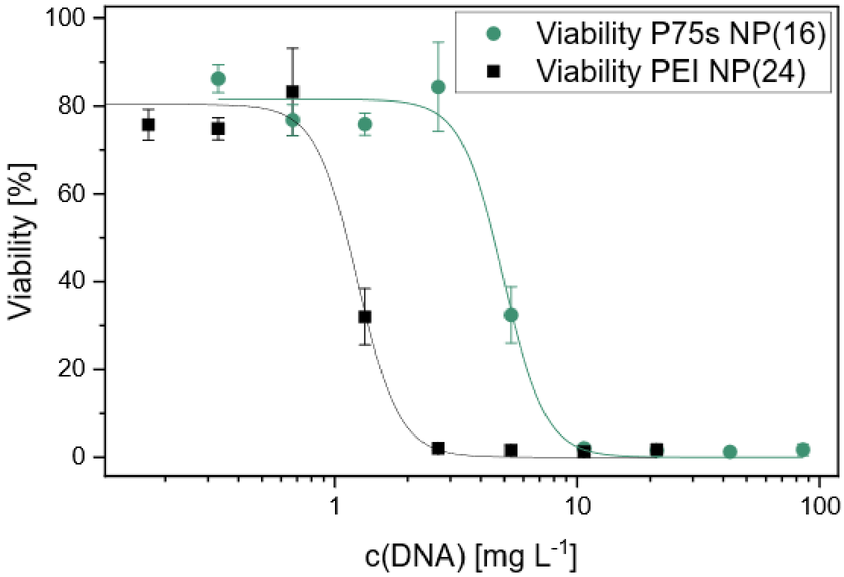

Figure S5: Percentage of cell viability of mouse L292 fibroblasts determined via the MTT assay for P75s and linear PEI 25kDa at set N/P ratios against the concentration of the polyplex (measured as the DNA concentration c(DNA)) used for polyplexation. Data points for cell viability under variation of c(DNA) (bottom right) were fitted applying the Hill equation.

References

(1) Lehnen, A.-C.; Kurki, J. A. M.; Hartlieb, M. The Difference between Photo-Iniferter and Conventional RAFT Polymerization: High Livingness Enables the Straightforward Synthesis of Multiblock Copolymers. *Polym. Chem.* **2022**, *13* (11), 1537–1546. https://doi.org/10.1039/D1PY01530C.

(2) Fulmer, G. R.; Miller, A. J. M.; Sherden, N. H.; Gottlieb, H. E.; Nudelman, A.; Stoltz, B. M.; Bercaw, J. E.; Goldberg, K. I. NMR Chemical Shifts of Trace Impurities: Common Laboratory Solvents, Organics, and Gases in Deuterated Solvents Relevant to the Organometallic Chemist. *Organometallics* **2010**, *29* (9), 2176–2179. https://doi.org/10.1021/om100106e.

(3) White, L. A.; Hoyle, C. E.; Jönsson, S.; Mathias, L. J. Effects of Cis–Trans Isomerization on the Photopolymerization Behavior of N-Vinyl Amide Monomers. *Designed Monomers and Polymers* **2005**, *8* (4), 297–308. https://doi.org/10.1163/1568555054460042.

(4) Lehnen, A.-C.; Bapolisi, A. M.; Krass, M.; AlSawaf, A.; Kurki, J.; Kersting, S.; Fuchs, H.; Hartlieb, M. Shape Matters: Highly Selective Antimicrobial Bottle Brush Copolymers via a One-Pot RAFT Polymerization Approach. *Biomacromolecules* **2022**, *23* (12), 5350–5360. https://doi.org/10.1021/acs.biomac.2c01187.

(5) Navarro, G.; Sawant, R. R.; Essex, S.; Tros de ILarduya, C.; Torchilin, V. P. Phospholipid–Polyethylenimine Conjugate-Based Micelle-like Nanoparticles for siRNA Delivery. *Drug Deliv Transl Res* **2011**, *1* (1), 25–33. https://doi.org/10.1007/s13346-010-0004-0.

(6) Stephenson, F. H. 5 - Quantitation of Nucleic Acids. In *Calculations for Molecular Biology and Biotechnology*; Stephenson, F. H., Ed.; Academic Press: Burlington, 2003; pp 90–108. https://doi.org/10.1016/B978-012665751-7/50046-9.
